# Supplementary material for: Metabolic Profiling and Comparative Proteomic Insight in Respect of Amidases during Iprodione Biodegradation
Source: Microorganisms. 2023 Sep 22;11(10):2367. doi: 10.3390/microorganisms11102367 (PMC10608976; doi:10.3390/microorganisms11102367)
Supplement: Supplementary file 1 [file microorganisms-11-02367-s001.zip › microorganisms-2575489-supplementary.pdf]

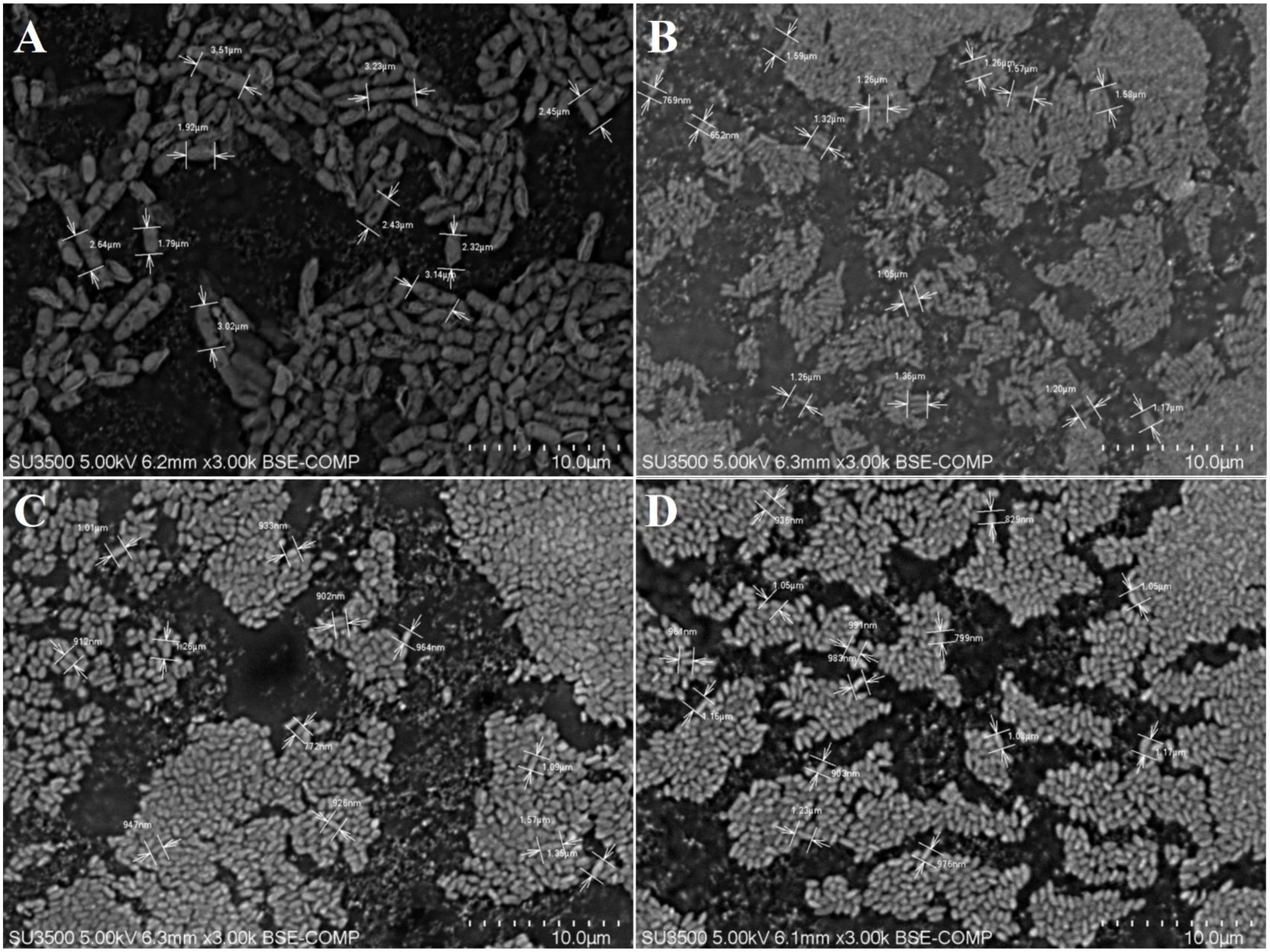
A
SU3500 5.00kV 6.2mm x3.00k BSE-COMP
10.0μm
B
SU3500 5.00kV 6.3mm x3.00k BSE-COMP
10.0μm
C
SU3500 5.00kV 6.3mm x3.00k BSE-COMP
10.0μm
D
SU3500 5.00kV 6.1mm x3.00k BSE-COMP
10.0μm

Figure S1.

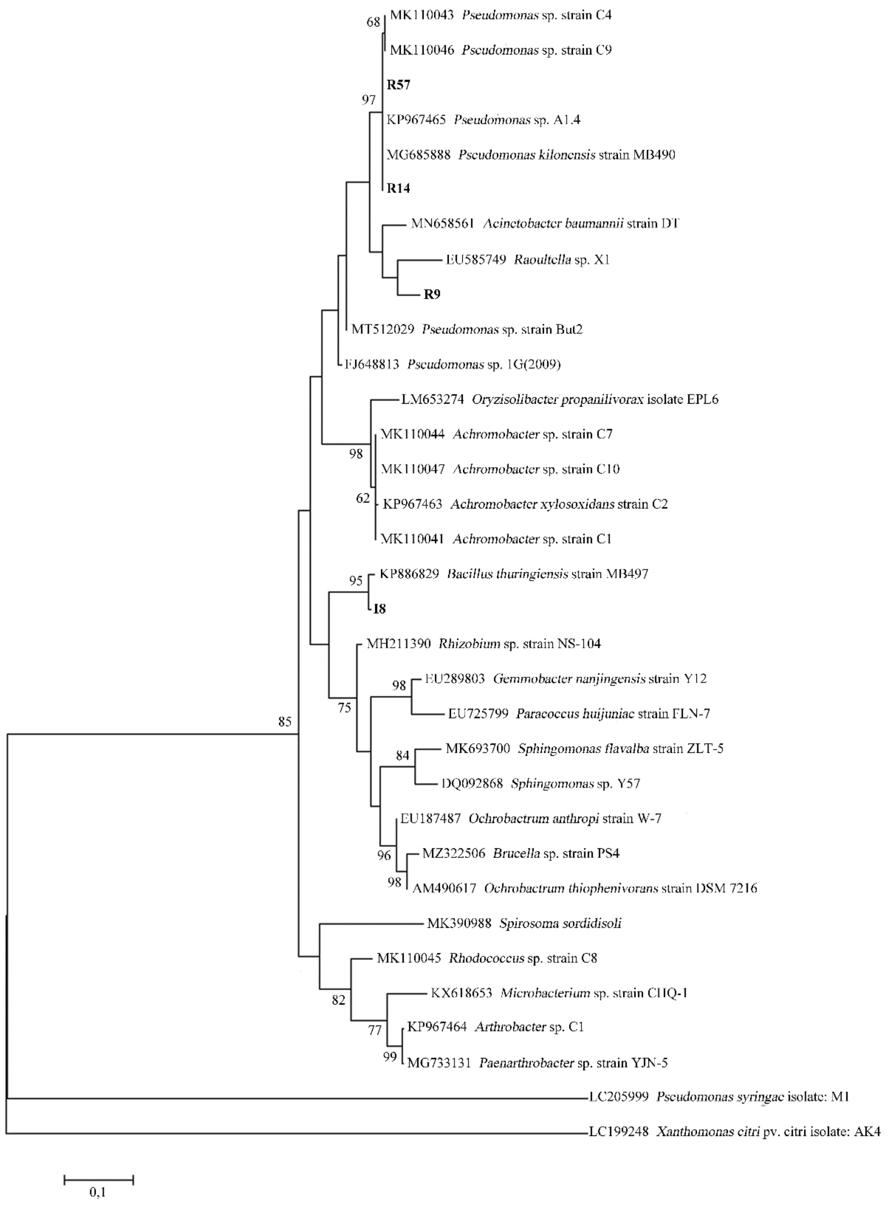
MK110043 Pseudomonas sp. strain C4
MK110046 Pseudomonas sp. strain C9
R57
KP967465 Pseudomonas sp. A1.4
MG685888 Pseudomonas kiloncnsis strain MB490
R14
MN658561 Acinctobacter baumannii strain DT
EU585749 Raoultella sp. X1
R9
MT512029 Pseudomonas sp. strain But2
FJ648813 Pseudomonas sp. 1G(2009)
LM653274 Oryzisolibacter propanilivorax isolate EPL6
MK110044 Achromobacter sp. strain C7
MK110047 Achromobacter sp. strain C10
KP967463 Achromobacter xylosoxidans strain C2
MK110041 Achromobacter sp. strain C1
KP886829 Bacillus thuringiensis strain MB497
I8
MH211390 Rhizobium sp. strain NS-104
EU289803 Gemmobacter nanjingensis strain Y12
EU725799 Paracoccus huijuniae strain FLN-7
MK693700 Sphingomonas flavalba strain ZLT-5
DQ092868 Sphingomonas sp. Y57
EU187487 Ochrobactrum anthropi strain W-7
MZ322506 Brucella sp. strain PS4
AM490617 Ochrobactrum thiophenivorans strain DSM 7216
MK390988 Spirosoma sordidisoli
MK110045 Rhodococcus sp. strain C8
KX618653 Microbacterium sp. strain CHQ-1
KP967464 Arthrobacter sp. C1
MG733131 Paenarthrobacter sp. strain YJN-5
LC205999 Pseudomonas syringae isolate: M1
LC199248 Xanthomonas citri pv. citri isolate: AK4
68
97
98
62
95
98
75
84
96
98
85
82
77
99
0,1

Figure S2.

Table S1.

Strain	IPR removal (%)	3,5-DCA (mg L^{-1})
N3	94.8	0.180 ± 0.040
I1	55.2	0.254 ± 0.030
I2	59.5	0.175 ± 0.010
I8	97.1	0.231 ± 0.030
I12	32	0.176 ± 0.050
M6	94.1	0.194 ± 0.010
M12	93.9	0.043 ± 0.010
R1	33.6	0.109 ± 0.040
R5	94.8	0.93 ± 0.050
R9	97.1	0.253 ± 0.010
R14	96.7	0.257 ± 0.040
R44	83.5	0.129 ± 0.070
R57	96.8	0.199 ± 0.090
C1	97.3	0.195 ± 0.050
C9	97.3	0.141 ± 0.020
Control	6.0	≤0.076 ± 0.000

The average values of two replicates are presented (n = 2); 3,5- DCA (mg L^{-1}): production of metabolite 3,5-dichloroaniline.

Table S2.

Strain	Biochemical test					Morphology		
	Amylase	Cellulase	Lipase	Protease	Gelatinase	Gram	Cell form	Colony
**I8**	+	+	+	+	+	+	Bacillus	Pink
**R9**	-	-	-	+	+	-	Bacillus	Yellow
**R14**	-	-	-	-	-	-	Bacillus	White
**R57**	-	-	-	+	+	-	Bacillus	White

+ : Positive reaction, - : Negative reaction

Table S3.

Enzyme	Response of strains			
	I8	R9	R14	R57
Control	-	-	-	-
Alkaline phosphatase	+	+	-	+
Esterase (C4)	+	+	+	+
Esterase lipase (C8)	+	+	+	+
Lipase (C14)	-	+	-	-
Leucine arylamidase	+	+	+	+
Valine arylamidase	-	+	+	+
Cystine arylamidase	+	+	-	-
Trypsin	-	+	+	-
α- Chymotrypsin	+	-	-	+
Acid phosphatase	+	+	+	+
Naphthol-AS-BI-phosphohydrolase	+	+	+	+
α-Galactosidase	+	-	-	-
β- Galactosidase	+	-	-	-
β-Glucuronidase	-	-	-	-
α- Glucosidase	+	+	-	-
β- Glucosidase	-	+	-	-
N-acetyl-β-Glucosaminidase	-	-	-	-
α- Mannosidase	-	-	-	-
α- Fucosidase	-	-	-	-

+ : Positive reaction, - : Negative reaction

Table S4.

Strain	Most closely related strain (NCBI Accession Nº) [a]	Identity (%)	Accession Nº
I8	*Priestia aryabhattai* strain B8W22 (NR_115953.1)	99.2	OM993327
R9	*Stenotrophomonas rhizophila* strain IHBB 9245 (KU921558.1)	97.9	OQ174722
R14	*Pseudomonas vancouverensis* strain DhA-51(NR_041953.1)	99.6	OM993328
R57	*Pseudomonas vancouverensis* strain LMG 20222 (NZ_LT629803)	99.6	OM993329

(a) Based on partial sequencing of 16S rRNA gene and comparison with those present in GenBank database from National Center for Biotechnology Information (NCBI) by using BLAST.

Table S5.

Up-regulated		Down-regulated		IPR Treatment*	
**M. Pathway****	**N°**	**M. Pathway****	**N°**	**M. Pathway****	**N°**
Total metabolic pathways	17	Total metabolic pathways	11	Total metabolic pathways	37
Biosynthesis of secondary metabolites	8	Microbial metabolism in diverse environments	4	Biosynthesis of secondary metabolites	18
Ribosome	7	Biosynthesis of cofactors	3	Microbial metabolism in diverse environments	13
Biosynthesis of amino acids	5	Biosynthesis of secondary metabolites	3	Biosynthesis of amino acids	10
Microbial metabolism in diverse environments	5	Carbon metabolism	2	ABC transporters	8
Glycine, serine and threonine metabolism	3	Pyruvate metabolism	2	Carbon metabolism	8
Oxidative phosphorylation	3	Two-component system	1	Propanoate metabolism	6
Biosynthesis of nucleotide sugars	2	Oxocarboxylic acid metabolism	1	Valine, leucine and isoleucine degradation	5
Carbon metabolism	2	Pantothenate and CoA biosynthesis	1	Pyruvate metabolism	5
Cysteine and methionine metabolism	2	RNA degradation	1	Quorum sensing	4
Lipopolysaccharide biosynthesis	2	Cationic antimicrobial peptide (CAMP) resistance	1	Nucleotide metabolism	3
Biosynthesis of cofactors	2	Arginine biosynthesis	1	Phenylalanine, tyrosine and tryptophan biosynthesis	3
Pyruvate metabolism	2	Glutathione metabolism	1	Glycolysis / Gluconeogenesis	3
Bacterial secretion system	2	beta-Lactam resistance	1	Glutathione metabolism	3
ABC transporters	1	Dioxin degradation	1	Histidine metabolism	3
Pyrimidine metabolism	1	beta-Alanine metabolism	1	Methane metabolism	2
Phenylalanine, tyrosine and tryptophan biosynthesis	1	Fatty acid biosynthesis	1	2-Oxocarboxylic acid metabolism	2
Naphthalene degradation	1	Degradation of aromatic compounds	1	Arginine and proline metabolism	2
Tyrosine metabolism	1	Biosynthesis of amino acids	1	Fatty acid metabolism	2
Biotin metabolism	1	Butanoate metabolism	1	Valine, leucine and isoleucine biosynthesis	2
Fatty acid metabolism	1	Ribosome	1	Glyoxylate and dicarboxylate metabolism	2
Glyoxylate and dicarboxylate metabolism	1	Propanoate metabolism	1	Aminoacyl-tRNA biosynthesis	2
DNA replication	1	Biotin metabolism	1	Phenylalanine metabolism	2
Galactose metabolism	1	Xylene degradation	1	Alanine, aspartate and glutamate metabolism	2
Histidine metabolism	1	Pyrimidine metabolism	1	Pantothenate and CoA biosynthesis	2
Degradation of aromatic compounds	1	ABC transporters	1	Ribosome	2
Chloroalkane and chloroalkene degradation	1	Purine metabolism	1	Purine metabolism	2
Citrate cycle (TCA cycle)	1	One carbon pool by folate	1	Fatty acid biosynthesis	2

Lysine biosynthesis	1	Benzoate degradation	1	Flagellar assembly	1
Fatty acid biosynthesis	1	Fatty acid metabolism	1	Pyrimidine metabolism	1
Oxocarboxylic acid metabolism	1	Alanine, aspartate, and glutamate metabolism	1	Acarbose and validamycin biosynthesis	1
Phenylalanine metabolism	1	Nucleotide metabolism	1	Cysteine and methionine metabolism	1
Homologous recombination	1	Oxidative phosphorylation	1	Benzoate degradation	1
Glycolysis / Gluconeogenesis	1			Inositol phosphate metabolism	1
Fatty acid degradation	1			Monobactam biosynthesis	1
Arginine biosynthesis	1			Novobiocin biosynthesis	1
Protein export	1			RNA degradation	1
Mismatch repair	1			Streptomycin biosynthesis	1
Valine, leucine and isoleucine degradations	1			Lysine biosynthesis	1
Nucleotide metabolism	1			Biosynthesis of nucleotide sugars	1
Quorum sensing	1			Tyrosine metabolism	1
Purine metabolism	1			Bacterial chemotaxis	1
Peptidoglycan biosynthesis	1			Two-component system	1
Amino sugar and nucleotide sugar metabolism	1			Lipopolysaccharide biosynthesis	1
Methane metabolism	1			Taurine and hypotaurine metabolism	1
Valine, leucine and isoleucine biosynthesis	1			Degradation of aromatic compounds	1
Seleno compound metabolism	1			beta-Alanine metabolism	1
				Glycine, serine and threonine metabolism	1
				Arginine biosynthesis	1
				Tryptophan metabolism	1
				Butanoate metabolism	1
				C5-Branched dibasic acid metabolism	1
				Citrate cycle (TCA cycle)	1
				Polyketide sugar unit biosynthesis	1
				Nitrogen metabolism	1
				O-Antigen nucleotide sugar biosynthesis	1
				Pentose phosphate pathway	1

IPR Treatment*: Solely expressed on IPR Treatment; M. Pathway**: Metabolic pathways by KEEG; N°: number of involved proteins
